# Turning over New Leaves

**Published:** 1889-01-19

**Authors:** 


### Turning Over New Leaves.

(Books for Review should be sent to The Editor. The Lodge, Porchester Square, W.)

### The Alehouse and the Hospital.*

Dr. Schofield has not the pen of an accomplished novelist; but he has the pen of an earnest man, which is very much more to his purpose. Looking on "Queen Anne's Hospital" simply as a story, we might demur to the introduction of certain incidents which, though doubtless taken from real life and not lacking in intrinsic interest, have no bearing on the principal narrative. Also we might protest against the improbability of the central interest of the story—the heroine's assuming male dress, and wearing it for nearly three years, during which time she pursues her medical studies at Queen Anne's Hospital, and poses as the husband of her own mother. We are not of those who think that every incident in a novel must have "gone through the formality of having actually occurred," as Balzac puts it; that is essential for newspapers, but not for artistic fiction. But the novelist achieves success only when he convinces his readers that the incidents he narrates might reasonably and naturally occur; when he makes them feel that, in the given circumstances, the incidents must inevitably have happened as he tells them, he attains a triumph.

* "Queen Anne's Hospital: Its Senators and Sufferers." By A. T. Schofield, M.D., Author of "Travels in the Interior," "Another World; or, The Fourth Dimension." London: Swan Sonnenschein and Co., Paternoster Square.

But Dr. Schofield's book does not profess to be a mere story, told for the story's sake; it is a statement, and a proffered solution, of one of our most difficult national troubles—the drink question.

The connexion between the alehouse and the hospital is this—that drunkenness is the cause of most of the accidents that fill the casualty wards of our hospitals, besides laying the foundation of diseases that ultimately lead to the same bourne; therefore, that if the traffic in liquor were suppressed, or sensibly lessened, there would be a corresponding reduction in the demand on hospital accommodation, and in proportion a reduction of hospital expenditure and hospital debt. Dr. Schofield is very severe, perhaps unjustly severe, on those great brewers who head the subscription lists for the benefit of hospitals, in which the sufferers are the victims of the drink these philanthropists have made and sold. We say unjustly severe, because the beer sent out by these great firms is generally pure and good, and it is bad beer, manufactured by makers whose names are hardly known to the world, and who have no feeling of business honour, that causes the greater part of our national drunkenness and its attendant evils.

Conceding this point, however, we are at one with Dr. Schofield in his declaration that many of our hospital patients are sent there by drunkenness, either their own or others', and that the way out of the difficulty is to be found, not in suppressing public-houses, but in making them more deserving of their name. A public-house should not be a drinking-shop, but a place of public refreshment, a place which it should not be a disgrace for a respectable woman, if not a respectable man, to enter. The mistake hitherto has lain in making refreshment houses one of two kinds—places where no alcoholic drinks can be obtained, and places where hardly anything else can be got, and having the latter far the more attractive of the two. Anyone who compares the average dull, if not dirty, coffee-shop, with the gay, well-lighted gin-palace, can hardly wonder at a weary and perhaps worried man preferring the latter. Yet it is desirable that he should choose the former, where he will receive no beverage that will do him harm, and where he can get food as well as drink. How is this to be managed? Here is Dr. Schofield's suggestion, given in his heroine's own words to her brewer husband, the owner of forty two public-houses :—

"Transfer the whole of them into light, cheerful working-men's clubs, put comfortable seats in them all, and break down the different partitions, so as to make the places thoroughly comfortable. Leave the bars as they are, and sell over them plenty of good tea, coffee, and cocoa, milk, hot beef-tea, tobacco—I would let the men smoke—and, I think, beer as well, but no wine or spirits whatever."

"Beer, wife? Not our beer?" asks the husband.

"No, not your strong ale, of course. But that light lager beer we get for ourselves. You told me all about it the other day. . . . This is what you told me: that you could brew a light sparkling ale, with not more than four per cent. of alcohol (which you told me was little more than was often found in ginger beer), that could not intoxicate anyone."

Now this is a reasonable, moderate, and feasible plan. The British soul hates coercion; trying to make a man sober by Act of Parliament is a very likely way to make him drunk. Not that we object to a reasonable amount of legislative action, on the contrary we are thankful to record a very considerable diminution in casualties at the London hospitals on Saturday nights since the Early Closing Act came into force. Still an absolute "Thou shalt not" on any subject whatever is apt to produce in the mind of perverse humanity a very determined "I will." Therefore "let the poor man have his beer," and the rich man too, but let it be of such a character as to do him no harm.

It is interesting to know that an experiment somewhat similar to the one suggested here has been tried in the village of Hampton Lucy, in Warwickshire. The vicar, the Rev. O. Mordaunt, bought up the public-houses in the place, and supplied them with good pure light beer in lieu of the heavy, adulterated, salted stuff that is usually sold in village alehouses. The result has been no diminution in the sale or consumption of beer, as the returns show, but an absolute cessation of drunkenness, with its contingent evils. From the profits of these public-houses the vicar has been able to improve the water supply and lighting of the village, but these results, though eminently pleasing, have nothing to do with our present argument. They are the voluntary act of a man who does not wish to make any personal profit out of a philanthropic experiment, and we have to deal with public-houses as properties whose owners wish to make a living out of them.

And on this point we think Dr. Schofield is timid. He represents Kenneth Falconer as losing £15,000 the first year of the existence of his regenerated public-houses, and admits only that they began to pay a little afterwards. We are of opinion that the retailing of tea, coffee, and cocoa leaves as great a profit to the retailer as the sale of alcohol. One might call into evidence on that point the great success of the Aerated Bread Company. While it existed only as a company for the manufacture and supply of bread the A. B. C. had but a limited success. The foundation of its financial prosperity was the establishment of the now well-known refreshment shops all over London. These speedily became popular, for they met a felt want, and they have raised the shares some hundreds per cent. in value. For there are many people who do not want to get drunk. They prefer wholesome refreshment when they can get it, and formerly were driven to stimulants simply from the difficulty of getting anything else easily, and at a moderate price. It would be interesting to know how much the establishment of these and similar shops has done to lessen drunkenness in London.

But if beer be desired, light beer will give an absolutely greater profit to the brewer than strong ale. It costs less to make, and, in all probability, will be consumed in larger quantities, while it can be sold at a greater profit. It may be said that this offers no remedy for the waste of money in liquor. It does not, directly; but it must be remembered that though drunkenness, as a form of extravagance, is to be regretted; drunkenness, as a vice in itself, is more deplorable still, leading, as it does, to moral and social evils of the most serious kind. We must allow for amusement as being one of the essential needs of man's nature, and we must allow also that, for the working classes, the public-house is almost the only place where amusement and company can be got, and, that being so, the first attempt of the reformer—better even than that of providing counter-attractions—will be to render harmless the places which have the charm of old association. Dr. Schofield's book offers a simple, rational, and practical cure for a deep moral disease, and as such we prize it, and we hope that the suggestions it contains will be widely carried out.

### Messrs. Cassell's Publications.*

"Commodore Junk" is a vigorous, well-written, and in parts pathetic tale of the old Marryat stamp that boys love, but touched with a deeper and tenderer humanity than the older writer usually displayed. Mr. Manville Fenn is always readable and wholesome, and he has succeeded in making the character of Mary Dell, in spite of her most unwomanly trade of pirate, both consistent and sympathetic.

The yearly volumes of "Bo-Peep" and "Little Folks" are intended for children at different stages of their growth, and their simply-told stories and catching rhymes should give no little information to those young people who are fortunate enough to get them.

The "Quiver" requires no praise; its wide popularity is the best testimonial it could possess. The stories are all interesting, the poems are above the average, and the numerous religious and descriptive papers are by well-known authors.

### Books for Boys and Girls.*

"Wonderful Adventures" contains enough of incident and romance to have supplied Mayne Reid with the material for half-a-dozen stories; and though the fights, imprisonments, and hairbreadth escapes are not treated so sentimentally as they were by the gallant captain whom we loved in our youth, they have the advantage of being strictly true. The price of this book, which has a number of good illustrations, is only a shilling; and the boy who doesn't enjoy it should be ashamed of himself.

"Claimed at Last" and the story which is bound up with it, "Roy's Reward," are good wholesome reading for children. The style is simple and straightforward, and the more striking adventures are told with considerable dramatic power.

"School Girls" is almost too didactic—a fault almost inevitable in dealing with such a subject. Girls do not get into scrapes like their brothers; their life has more calm on the surface, and, as a rule, more emotional depth. Therefore any story dealing with them must be taken up with sayings rather than doings. The aspirations of the more thoughtful pupils at a girls' school are depicted in this volume with fidelity, if not with vivacity, and it will doubtless meet with acceptance among girls similar to those it treats of.

* All these books are published by Messrs. Cassell & Co. Lim., London.

